# Open source challenges for hospital information system (HIS) in developing countries: a pilot project in Mali

**DOI:** 10.1186/1472-6947-10-22

**Published:** 2010-04-16

**Authors:** Cheick-Oumar Bagayoko, Jean-Charles Dufour, Saad Chaacho, Omar Bouhaddou, Marius Fieschi

**Affiliations:** 1 DER Santé Publique, Faculté de Médecine, Pharmacie et d'Odonto-Stomatologie, Bamako, Mali; 2 Laboratoire d'Enseignement et de Recherche sur le Traitement de l'Information Médicale, Faculté de Médecine, Université de la Méditerranée 27, boulevard Jean Moulin 13385 Marseille Cedex 5, France; 3 Unité de Veille Technologique et de Développement Logiciel, Centre Hospitalier Ibn Sina de Rabat, Maroc; 4 Hewlett Packard Enterprise Services and Department of Veterans Affairs, San Diego - CA, USA

## Abstract

**Background:**

We are currently witnessing a significant increase in use of Open Source tools in the field of health. Our study aims to research the potential of these software packages for developing countries. Our experiment was conducted at the Centre Hospitalier Mere Enfant in Mali.

**Methods:**

After reviewing several Open Source tools in the field of hospital information systems, Mediboard software was chosen for our study. To ensure the completeness of Mediboard in relation to the functionality required for a hospital information system, its features were compared to those of a well-defined comprehensive record management tool set up at the University Hospital "La Timone" of Marseilles in France. It was then installed on two Linux servers: a first server for testing and validation of different modules, and a second one for the deployed full implementation. After several months of use, we have evaluated the usability aspects of the system including feedback from end-users through a questionnaire.

**Results:**

Initial results showed the potential of Open Source in the field of health IT for developing countries like Mali.

Five main modules have been fully implemented: patient administrative and medical records management of hospital activities, tracking of practitioners' activities, infrastructure management and the billing system. This last component of the system has been fully developed by the local Mali team.

The evaluation showed that the system is broadly accepted by all the users who participated in the study. 77% of the participants found the system useful; 85% found it easy; 100% of them believe the system increases the reliability of data. The same proportion encourages the continuation of the experiment and its expansion throughout the hospital.

**Conclusions:**

In light of the results, we can conclude that the objective of our study was reached. However, it is important to take into account the recommendations and the challenges discussed here to avoid several potential pitfalls specific to the context of Africa.

Our future work will target the full integration of the billing module in Mediboard and an expanded implementation throughout the hospital.

## Background

### Why this study is important?

The implementation of Clinical Information Systems (CIS) is key to the production of quality care, adequate management of rare resources and productivity. A recent study has revealed an objective correlation between the degree of adoption of technologies in healthcare and reduction of complications and mortality in hospitals [1]. This is clear evidence that a real return on investment for these systems is possible. Healthcare IT is a necessity that is imposed on all the countries of the North and South alike. However, a critical question arises and is yet to be answered. Namely, given the differences in financial, technological, and human resources, should developing countries consider a different strategy to achieve implementation and adoption of healthcare IT? The implementation of information systems can succeed if two main conditions are met, and these both come with a financial burden:

(1) A rigorous and consistent organization of the actors and processes of care in which they are involved. Without this organizational approach any attempt to computerization is likely to fail.

(2) A clear choice for the establishment of infrastructure (hardware and software) which always requires substantial financial investment.

Although costly in human, organizational, and structural resources, the first condition appears to be available to everyone as long as the hospital management is informed, tenacious, thorough and methodical. The concept of process is not always clearly identified (probably even less in the South than in the North) and the complexity of care processes when combined with inadequate management of these processes is a source of non-quality, and of costly and avoidable medical errors. Within a hospital, implementation of a CIS is based on the computerization of care processes as well as of support processes (administrative, accounting, logistics, etc.) to ensure coherence, feasibility and effectiveness of the clinical and business activities of the institution.

To meet the second condition, the method adopted in the North is mostly based on the purchase of software available in the marketplace. Most hospital entities no longer develop their own solutions in-house. This response is problematic for developing countries at two levels. First, they do not have the financial resources to acquire a commercial CIS. Second, they do not have the same culture and organization that are implicitly or explicitly imposed and implemented as part of the commercial solutions coming from the developed countries and for which these applications have been developed and tested.

Several studies have examined the North-South transfer of information systems, including an important one by Richard Heeks [2]. He concluded that the information systems that succeed are those that best incorporate the key technical, social, and organizational environment aspects in which they are implemented. Heeks also noted that the failures are mainly due to a North-South transfer of information that does not take into account the context, or the local attitudes towards modernization and rationalism.

Therefore, developing countries face an important risk of being excluded from the path towards the computerization of healthcare facilities or systems, even as these are more necessary than ever to better manage the quality of care and the limited resources available to developing countries.

If commercial software packages seem out of reach for many poor countries, the fundamental principles behind the emergence of Open Source software and the acquisition cost of software, often free of charge, is a great opportunity for developing countries. Moreover, as stressed by Didier Lamouche [3], the interest of Open Source is also in its ability to allow firms and nations to possess and better manage their information systems.

Our article aims to analyze this particular situation while taking into account the emergence of Open Source software, and to propose a suitable and accessible development strategy that can be mastered by the South. Today, to the best of our knowledge, no country in French speaking Africa does possess a computerized information system that is adapted to the challenges of healthcare. In contrast developing countries of Latin America or other countries in Asia have made significant progress toward the computerization of healthcare processes in part through the use of Open Source software [4].

We will try to understand the opportunity of the Open Source movement in healthcare In particular; we report our experience with the use of Mediboard Open Source HIS at the Hospital Mère-Enfant le "luxembourg" in Mali.

We focus our remarks on hospital information systems that represent a clear and pressing need for developing countries, even if other applications, such as systems to aid in decision-making, to support HIV/AIDS care (i.e, OpenMRS, http://openmrs.org/wiki/OpenMRS), public health reporting, or clinical research are not of lesser importance. For example, the use of technologies such as portable PDAs in epidemiological surveillance is an interesting opportunity worthy of study as demonstrated by Yu P and al [5].

### The rationale of "Open Source" software

Mainly based on the sharing of source code and the collaborative development by the users themselves, Open Source software in the developed and developing countries has seen a steep increase over the last ten years. An example of this development is Linux which has now a significant market share of Operating Systems (Linux in 1997 accounted for 1% of the server market against 30% in 2007). Concerns about lack of standardization and security in Open Source software have been expressed for a long time and have limited their use in production systems. These concerns are now disappearing, as demonstrated by several studies [6]. It is likely that Open Source is reaching its maturity phase. Several nations have already mandated the use of Open Source in government agencies, as for instance in Brazil and South Africa. A recent bill has passed in the US senate aiming at the same (Jay Rockfeller - April 23, 2009).

Table 1 shows usage of Open Source software in Europe and North America according to a Forrester report [7].

**Table 1:** Usage of Open Source software in Europe and North America according to Forrester [5]

Web Infrastructure	76%
Server Operating Systems	76%
Developer Tools	66%
Network Infrastructure	42%
Databases	42%
Business Applications	9%

The basic philosophy of Open Source is not only to propose, whenever possible, free software for users, but also to give access to the source code.

From an economic point of view, the Open Source helps create a new form of market and economy. Open Source also creates profitability of investments in software development. Indeed, the development of the Open Source market is driven by traditional information technology enterprises and by service companies specialized in Open Source software and associated services.

**Figure 1:**
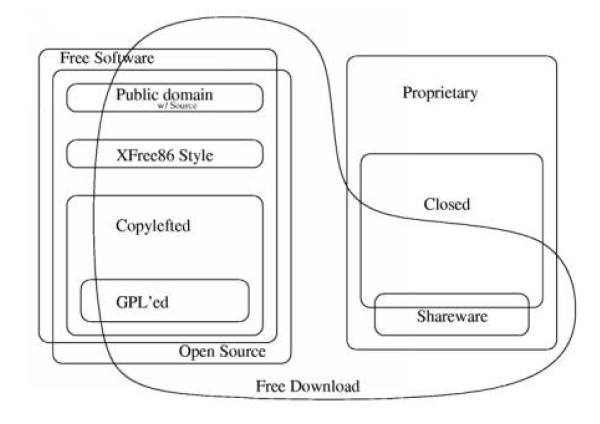
**Chao-Kuei's Diagram: different categories of software (**[6]**)**.

The Open Source software, similar to freeware, is very often free of charge but, unlike the commercial product, offers access to the source code. Some Open Source software are marketed by companies as "distribution." The conditions of use are specified in the license notices; the best known of them is the GPL (General Public License/GNU Public License). Figure 1 presents a scheme that makes it easy to locate the various categories of software. Table 2 gives a few characteristics of these different software.

**Table 2:** Characteristics of the categories of software

	Freeware	Commercial Software	Open source Software
**Source Code Provided**	NO	NO	YES

**Modification Allowed**	NO	NO	YES

**Redistribution Allowed**	YES	NO	YES

**Free Access**	YES	NO	YES/NO

For proprietary software, the threat of Open Source certainly comes from its very low cost, but also because it sometimes aims to define new standards for the industry (such as Linux, PHP, Perl and Apache). Sometimes, it does reach that goal.

The Open Source model has interesting properties for clients and also for suppliers and developers. For the customer, it ensures technology transparency and transfers or eliminates the cost of licensing services. As a result, one can develop a tailor-made product and benefit from a broad network of contributors.

For the small and medium enterprise, it offers a strategy for interesting Research and Development as it allows to draw from a large pool of software packages and benefits from the quality monitoring feedback of the community of developers and users. Thus, the deployment of Open Source solutions does not mean introducing static tools; as the specific needs of users can be developed and integrated without the obstacles brought by intellectual property. Any service-oriented business can offer specific development on Open Source infrastructure and the only limitation is the acquisition of technological expertise and resources necessary for the development. What is true for a small enterprise is also true for countries in the South. This is very important and makes the software accessible to the poorest.

Figure 1- Chao-Kuei's Diagram: different categories of software ([8])

Table 2. Characteristics of the categories of software

If the acquisition of Open Source is less expensive, there should however be some allowance in terms of integration and support. This aspect must be taken into account in any implementation strategy. Thus, when commercial entities sell Open Source software, they replace the cost of the license by the cost of service.

Consequently, when evaluating the pros and cons, the most decisive criteria for the choice between Open Source and commercial software cannot only be the economic argument in favor of Open Source software. Other factors worth considering include:

• The non-availability of the source code is a security risk that is often mentioned. In fact, the weaknesses exploited by hackers in recent years do not support that argument and even seem to show otherwise [9]. The availability of the source code for the greatest number via a community of developers and users can help prevent or quickly and effectively address the vulnerabilities exploited by hackers.

• The graceful evolution of an Open Source product as well as its durability is also a source of doubt. It may be noted that the risk of graceful evolution of software exists for both Open Source and proprietary software. There are many examples of commercial companies, sometimes the most important ones, who have stopped their activities in the medical field, or interrupted the development of a product, or, for reason of bankruptcy, put their customers in difficult situations. In such situations, organizations that use proprietary software have less option than those who have adopted for Open Source solutions. The latter ones can more easily turn to third-party companies to ensure continuity of service.

### The Development of Open Source Software in Healthcare

Recent years have seen a steep rise of Open Source applications in healthcare. However, this is not a new phenomenon. In the 1970s, Octo Barnett made available the source code of an ambulatory medical record system http://costar.net/costar.htm. More recently, the healthcare division of the Department of Veterans Affairs has embraced the same movement by putting in the public domain its complete clinical information system called VistA http://www.worldvista.org/World_VistA_EHR.

In the domain of healthcare there are now more than a hundred free open source applications. In addition to these free software packages, tools like Google Maps for example, can be interfaced with others software packages to improve the visualization of information. These types of systems can measure the progression of diseases and the effectiveness of various interventions [10].

An Open Source software community can benefit from the sharing of tools available but it is also the bearer of developments that meet and promote international standards such as architecture, data representation, terminology and interoperability standards [11]. Often community members behind Open Source software are actively involved in the development and adoption of open standards for a global information technology [7].

Many developing countries are experimenting with Open Source and the phenomenon is growing [12]. These developments are diverse and cover the management of general and multi-disciplinary medical records, as well as more specialized information systems related in particular to public health issues like HIV/AIDS surveillance [13].

Therefore, the Open Source applications are a superior information system option, due to their open methodology and their cost. This opportunity has been identified by several consortia that have formed on this issue: the Open Source Health Care Alliance (OSHCA, http://resmedicinae.sourceforge.net/oshca/index.html), the American Medical Association Open Source Working Group (AMIA, http://www.amia.org and the Open Health Tools (OHT, http://www.openhealthtools.org) who has several projects including the development of an open source tool (workbench) for managing the rich ontology SNOMED CT [14].

## Methods

### Mediboard Open source software package selected for the pilot project in Mali [15]

Several Open Source applications exist today in the field of HIS [16,17]. Mediboard application package has been verified to also be on the SourceForge (a public directory of open source software accessible at http://sourceforge.net/projects/mediboard/). The selection of Mediboard for the project in Mali was made because the software was:

• Implemented at several sites and used in practice. His metrics of use in France (see Table 3) have positively influenced our choice for implementation in Mali.

**Table 3:** Anonymized and Aggregate Overview of Mediboard deployments in France

Facilities	08
**Users**	1200

**Beds**	510

**Surgery rooms**	55

**Patients**	430000

**Visits/Consultations**	520000

**Hospital Stay**	150000

**Interventions**	140000

**Documents**	280000

**Attachments**	130000

**Figure 2:**
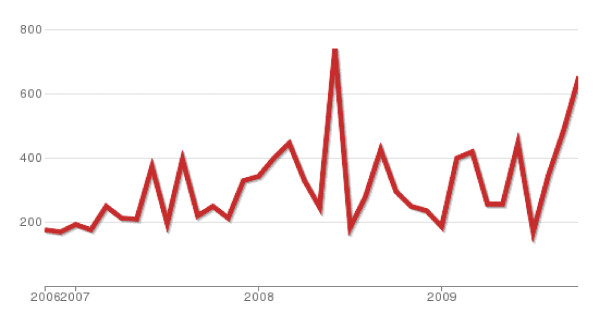
**Number of commits made to the Mediboard project source code each month**.

• Supported by a large and active community (guarantee of durability) as evidenced by data from the web site "ohloh". This site also provides the application package data which is illustrated in Figure 2

Table 3-Anonymized and Aggregate Overview of Mediboard deployments in France

### Implementation Approach

The implementation was preceded by a period of user training which included basic computer skills, because many were in need of updating their basic knowledge of how to use computers.

The creation of an intranet for information dissemination was also helpful before the start of the implementation.

The application was installed on two Linux servers: the first was used for testing and validation of different modules as well as for the training of users, and the second hosted the production implementation of the HIS.

All the features covered by Mediboard can be installed at the same time. To enable a particular function only requires its activation in the management module accessible to the system administrator.

This implementation was done on a modular and incremental basis, following a set of priorities defined by a special steering committee of the hospital put together for this purpose.

After each test, the module was validated after discussion with users to determine if the function is adapted to their needs. After user validation, a training plan was put in place and production did not begin until users felt completely comfortable working with the tool. A process of ongoing training was then established at the hospital.

Users did have 24/7 access to the technical support team for assistance. This support was provided by a crew of young doctors and computer information system specialists based at the hospital "Mere -Enfant", and that has been working in the field of telemedicine in Mali for 9 years.

### Evaluation

The first step dealt with the ergonomics of the system and the overall end-users view of the whole process of computerization.

Regarding the ergonomic aspects, we collected users' opinion about the following criteria: the system's usefulness, usability, user-friendliness and timely accessibility. Using those parameters, we asked them to answer the following questions:

• Does the use of the system lead to a waste of time?

• Is the time spent using the system compatible with the tasks and the workload of the users?

• Do users prefer to enter their own medical data or delegate the task to a third party individual (e.g., clerk)?

• Does the system increase the reliability of data?

• Does the use of the system add value to your work?

• Has the quality of your work improved with the use of the system?

• Has the system changed the way you work?

• Have you been sufficiently (or adequately) trained to use the system?

• Are you ready to continue the experience with the deployment of new features?

• Do you think the system must be expanded to the whole hospital?

We used a questionnaire to collect written responses. Responses to evaluate the ergonomic aspects were based on a 4-point scale: 0 if the statement is completely false, 1 if the statement is negative, 2 if the statement is true and 3 if the statement is totally positive.

About the users' point of views on the system in general, there were "yes" or "no" questions with the possibility of inserting free-text comments.

This evaluation was conducted with 13 users including 8 doctors, 2 senior nurses and 3 administrative staff members.

The data were analyzed using Epi software date version 2.1.0.15 http://www.epidata.dk/download.php

## Results

### Preliminary Results

In large part, Mediboard meets the functional requirements as established by our expert team from the University Hospital of Marseille (Table 4) and validated by the global healthcare IT company Cerner http://www.cerner.com/public/. In fact, Mediboard meets 32 of the 36 functionality features of the in-take registration book. Thus, the compatibility rate is approximately 89%. We recognize that these requirements are not specific to developing countries; however, given the lack of African specific requirements, they provide a useful basis to evaluate Open Source alternatives.

**Table 4:** HIS Functional requirements of Marseille University Hospital versus Mediboard

Standard HIS features	Mediboard Open Source HIS Application
**Care Management**	

Registration	X

Appointments & Scheduling	X

Management of Movement (Transfers)	X

Care Plan Management	X

e-prescription (acts, medicine)	X

Nursing	X

Report and Mail Management	X

Logistics	X

Resource Management (stocks, human, materials)	X

Clinical Research, Epidemiology, Statistics and Education	X

Health Information Exchange	X

**Laboratory management (orders)**	

Pharmacy management	X

Imaging	X

Exploratory Procedures	

Emergency Department	X

Surgery Department	X

**Admin functions**	

Patient identity management	X

Outpatient Visits, Admissions, Stays	X

Bed Management	X

Evaluation of production activities (French PMSI Management)	X

Billing	X

**Facility Management**	

Access Management Rights/Entitlements	X

Activity Management	X

Medical Economics Management	X

Accounting and Record	X

Human resources	X

Equipment Management	X

Purchasing/Inventory	X

**HIS Environment Management**	

HIS Infrastructures	

Monitoring and Planning Tools	

Communication Management	X

Repositories and Terminology Management	X

**Other Features**	

Clinical Decision Support	

Digital Work Space	X

Data Warehousing	

Quality of Care Assessment	X

Moreover Mediboard is a French Open Source product in the field of hospital information systems based on web technologies. This is one of the key criteria that justifies its choice for the experiment set up in Mali to establish an HIS model adapted to countries in French speaking Africa. It has been indexed since 2005 in SourceForge and, today, it has the most active development community, according to published statistics. The functionality of the system, the possibility of an implementation directly in the French language, and the ease of configuration would make it a potential model for developing countries especially for those whose official medical language is French.

Figure 2 illustrates the level activity for Mediboard on data from, the Olho website http://www.ohloh.net that monitors the Open Source applications.

Table 4- Summarizes an analysis of the HIS functional requirements versus Mediboard Open Source.

The actual features implemented were as the follows:

**(a) Patient Administrative and Medical Record**, which covers the following items:

• Patient's Identity Management: MediBoard uses an auto-incremental numerical value system managed by the database server and stored in 4 bytes to handle more than 4 billion (2 ^ 32) identifiers. When interfacing with third party programs the identifier may accept other data type than an auto incremented value;

• Advanced search engine including results that are phonetically similar;

• Management of hospital stays;

• Admission, discharge, and transfer of patients;

• Creation of medical records;

• Management of duplicate files;

• Merging of records;

• Merging of hospital stays;

• Re-allocation of hospital stays;

• Production of documents based on models: admission forms, consents, information sheets, prescriptions;

• Electronic management of documents of all formats (e.g., images, pdf, etc.) with multi pages display;

• Management of medical history and allergies

• Shared annotations and alerts systems between health professionals and personnel of the organization.

**(b) Activities of the Health Facility**: The functions implemented deal with the planning of admissions and discharges of patients, coding of diagnoses and medical procedures, hospitalization planning (bed allocations, changes in services), quality management log (creation and tracking of incidents reports, electronic management of various procedures) and the master board of activities

**(c) Activities of the Practitioners: **this feature mainly focuses on the management of appointments and medical consultations and the daily and weekly master boards.

**(d) Infrastructure Management: **This module allows secured access management. Users can be grouped by department and function; advanced administration rights and permissions settings for users and ability to track the tasks performed in the system (reports by type and user) are also key.

**Figure 3:**
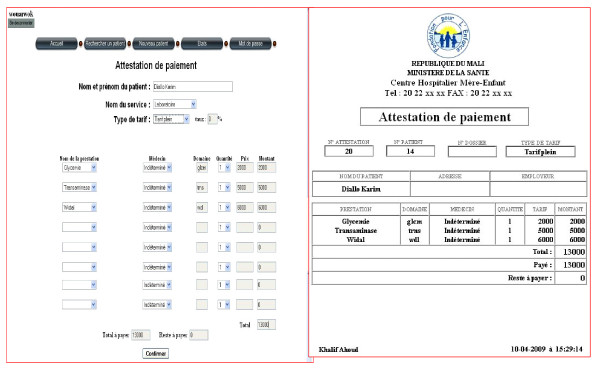
**Screenshot of billing module and receipt implemented by Local Team in Mali**.

**(e) Billing Module **(figure 3 is an example of a bill)**: **This is the only component that was fully developed by the local team in Mali because the one proposed by MediBoard was not adapted to the realities of Mali. There is a link between this module and the rest of the application platform but the challenge remains its full integration into MediBoard.

The implementation of these features began with the admissions office in charge of registering and recording the administrative data about the patient and reached progressively the general medical services, the obstetrics and gynecology and pediatrics units.

**Figure 4:**
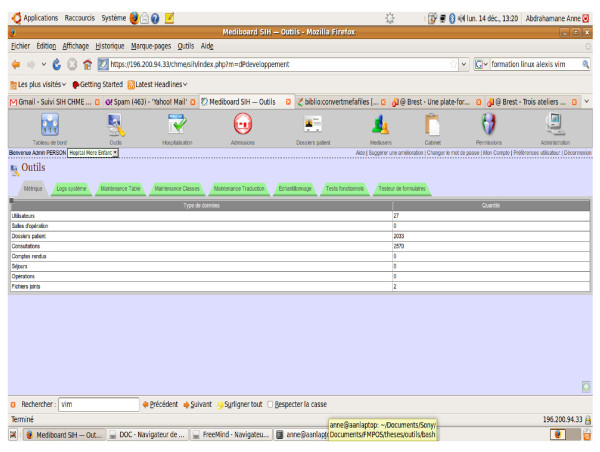
**Screenshot of Mebiboard HIS use in Hospital "Mère -Enfant" in Mali**.

In terms of computerization of Hospital Mere-Enfant, over a period of four months, we have captured over 2,754 medical records and provided 3,659 consultations with 13 users, all of whom are licensed physicians. The support for medical technical services (e.g., laboratory) is being provided (Figure 4 is the screenshot of Mebiboard HIS use in Hospital "Mère -Enfant"**)**.

Technically, the system maintenance and support is handled by the team of telemedicine of the hospital and has not posed any particular difficulty.

### Rating

#### (a) Ergonomic Data

Ergonomically the system is generally accepted by the users. Asked about the usefulness of the system, 77% of the users consider it very useful against 23% who simply confirmed its utility. We observed the same proportion regarding the ease of use aspect of the system. When it came to the question relating to user-friendliness, only 15% of respondents had a negative opinion of the system against an 85% approval rate. Finally, regarding access time, approximately 54% of users believed they can access the program very quickly, 31% think it was acceptable and 15% found it too long.

Table 5- Classification of the Users According to their Views on the System Globally

**Table 5:** Classification of the Users According to their Points of View on the Ergonomic Parameters of the System

Ergonomic Parameters Evaluation								
**Rating Criteria**	**Usefulness**		**User-friendliness**		**Ease of Use**		**Time to Access**	

	**N**	**%**	**N**	**%**	**N**	**%**	**N**	**%**

**NO**	8	61.5	4	30.8	2	29	0	0

**YES**	5	39.5	9	69.2	6	71	13	100

**Total**	13	100	13	100	8	100	13	100

N = Number of users; % = Percent

#### (b) Opinion of the Users of the System and the Method of Implementation

On the question whether or not the use of the system can result in a waste of the time, less than half of the users (or 39.5%) responded affirmatively whereas more than half of them (or 61.5%) believed that it saves time.

On the compatibility with tasks and workload, only three users formally stated that it was incompatible, one user remained undecided (i.e., shared opinion) and nine (or 69%) affirmed without any ambiguity that it is compatible.

Among the eight physicians who participated in the assessment dealing with entering clinical data in the system database just two prefere to delegate that task to an assistant or a medical secretary, the other six (or 71%) prefered to capture these data themselves. 100% of the users strongly believed that the system increased significantly data reliability.

Users also unanimously (i.e., 100%) asserted that the system added value to their work and that they have been well trained to use it.

Concerning the impact of the system on the quality of the work only one user thought that the system had no impact on improving the quality of his work, another one was undecided and eleven (or 84%) believed that the computer information system had has increased the quality of their work.

Regarding the changes in the work habits, ten users (or about 77%) believed the system brought innovations in how they performed their activities against 23% who said that it did not require any adjustment.

On the future prospects of the system, 100% of users said that they are ready to continue the experience with the deployment of new features. Also, users unanimously indicated that the implementation of the program must be expanded to all the departments of the hospital.

Table 6- Classification of the Users According to their Views on the System Globally

**Table 6:** Classification of the Users According to their Views on the System Globally

User's Point of View on the System																				
**Responses**	**Waste of time**		**Compatibility**		**Data entry**		**Data reliability**		**Work valorization**		**Work improvement**		**Change in workflow**		**Good training**		**Deployment of news features**		**System expansion**	

	**N**	**%**	**N**	**%**	**N**	**%**	**N**	**%**	**N**	**%**	**N**	**%**	**N**	**%**	**N**	**%**	**N**	**%**	**N**	**%**

**NO**	8	61.5	4	30.8	2	29	0	0	0	0	2	15.4	3	23.1	0	0	0	0	0	0

**YES**	5	39.5	9	69.2	6	71	13	100	100	100	11	84.6	10	76.9	13	100	13	100	13	100

**Total**	13	100	13	100	8	100	13	100	13	100	13	100	13	100	13	100	13	100	13	100

N = Number of users; % = Percent

#### Lessons learned from the pilot project in Mali

In light of our study, the first lesson we can develop is that it is neither utopian nor unrealistic to believe in the feasibility of a Hospital Information System in the developing countries despite the well known shortage of material and operating infrastructure. The economic argument no longer holds since there are Open Source tools adjustable at low cost by local teams with two advantages: the development of local expertise and small business enterprises.

The fear of the inconvenience arising from the use of freeware is not a strong argument either in today's environment because these tools have the same functionalities and security risks as the commercial ones. Also, we must note that they are used nowadays by many large institutions of the most industrialized countries of the world.

Thus, the priority must focus in convincing the hospital decision makers in the developing nations and involving them directly in the entire process so they can be aware of the efficient role of information technology in the healthcare system.

In this respect, the role of influential bodies such as the WHO which has already recommended by a resolution the development of information technologies and communication in the field of health is crucial [18].

We have also learned that the non-computerization is time wasted by practitioners locating and retrieving patients' records. At the hospital "Mere Enfant" we observed that the pressure from doctors on their assistants for finding these records fell with the implementation of the system. Also, the hospital management confirmed a valuable decrease in the purchase of paper data sheets for patients.

The economic and material aspects should not hide the cultural ones that can be in some cases a real barrier to the deployment and adoption of the system should we fail to pay attention to them.

Indeed, when it comes to starting a new activity or setting up new equipment, it is easy to be confronted with "ego" problems strongly linked to culture. For example, we tend to believe that any investment should start with the age order (i.e., from the oldest to the youngest) and this is considered as a great sign of respect in any social or professional sector. The concept of "Elder", therefore, becomes very important. They must always be the first to be served as it is always said "the first served are the best served." This element is taken very seriously in the Malian society and generally in all parts of Africa. Thus, it is easy for this kind of project to fail without any clear or apparent reason since people are usually reluctant to express openly these kinds of feelings. Still, this remains culturally a "taboo".

Also, we learned that using such a system is a source for quality medical information. For instance, the system required users to code the diagnoses of medical and surgical acts as soon as the decision to hospitalize or administer a care was made, thanks to a preconfigured system function.

Finally, on the maintenance issue, the challenge still remains with the development and release of new and stable versions of the system production tools. Indeed, despite the dynamism and constant activities in the Mediboard development community, we find that it takes some time to make recent released versions more stable. To meet this challenge, we initiated an ongoing dialog between the Mediboard and local Malian development teams so the latter can participate more actively in the engineering of the application.

## Discussion

### Difficulties of our study

During our research, we quickly came to realize the need for an implementation tool. The concept to utilize Mediboard, a French hospital information system model, emerged. It became clear that an adaptation study of this Open Source opportunity was required. William and Thierney studies demonstrated the feasibility of setting up a health information systems (HIS) if there is a suitable methodology in place [19,20].

Modifications were undertaken, which focused mainly on two programming functions:

• The patient administrative record module: at this level, the task consisted of the temporary deletion of certain data fields such as social security information which is not a valid personal information in Mali (i.e., does not exist), and the phone number that was not compatible with the Malian numbering format because of the number of digits. The number of digits in phone numbers in Mali is six (6) for landline and eight (8) for mobile phones, whereas in France it is nine (9) for both types.

• Billing: this is the part that has undergone a radical change in both substance and form. There is a huge difference between the French and Malian billing systems:

◦ First, unlike the French system, in Mali, the patient has to pay before any medical care is rendered regardless of the type of procedure requested;

◦ Second, in Mali, there is no health insurance coverage, therefore, patients pay for the services directly from outs-of-pockets. In other words, there is (health insurance company) reimbursement plan;

◦ Third, the currency (EURO), which the module was developed to recognize is different from that used in Mali (FCFA) and there was no built-in currency conversion utility;

◦ Considering all these factors, we developed a complete billing feature adapted to the context of our situation. Eventually, it will be integrated with the rest of the Mediboard platform.

### Barriers to Overcome

The potential of Open Source in the development of HIS is undeniable. However, there are many barriers to overcome in order to convince policy makers and users in developing countries. These barriers have already been studied in other countries such as Canada [21]. These barriers include:

• The resistance to change since most users and even the technicians do not have that culture.

• The problem of documentation: freeware often lack well-designed user manuals unlike the well-developed ones that usually come with proprietary software. But the revolution in the collaborative web is becoming an increasingly attractive alternative. For example, any documentation of Care2× is available on a wiki site http://care2x.org/wiki/index.php/Main_Page.

• The language barrier because almost all packages are developed in English without translation. For French speaking countries, this barrier is important, however an organized community could mitigate this problem.

• The absence of a single contact for free tools unlike for the commercial ones.

• Lack of financial support for the development of Open Source projects and the risk of losing any existing fund.

These barriers are not insurmountable given the importance of the application of Information Technology and Communication in the healthcare sector in developing countries. They can be circumvented by a professional support system taking into account the culture and the specific situation of these countries.

### Practical Recommendations

"Clever technology can help solve two big problems in health care: overspending in the rich world and under-provisioning in the poor world." - The Economist, April 16^th^, 2009.

The Open Source option should be taken seriously at the highest level of health businesses. The choice of Open Source must win by merit and not by opportunism and appeal of free. In addition, it must also fit into a well-defined business architecture and meet the same rigorous standards, security requirements and user needs. After analysis of several types of information systems in several countries of the South, Heeks recommended reducing the gap between concept and reality by applying what he called "local improvisation" and that we could translate as contextualization and implementation of the principle of reality. Heeks identified four areas of activity that help fill these gaps:

• Identify the organizational realities - this requires open communication and considering the local reality as legitimate.

• Improve local technical skills, including IT project management skills.

• Inform key players about the limitations of information systems, and about the methods of evaluation and integration used.

• Analyze the 'How' as well as the 'What' - the implementation plan should be as well thought as the technological solution itself.

The computerization of care processes in developing countries should not be abandoned for any reason whatsoever. As we noted in the introduction, the process of computerization requires a rigorous organization and an analysis of the existing processes; it enables their evaluation and seeks to improve them. Thus, a project of computerization can be seen as a catalyst to introduce a methodology that is either lacking or inadequate in the hospitals of these countries. It can help formalize the process and motivate the players to implement needed processes. Such a project is therefore a lever that managers of hospitals and clinicians need to promote a rationale and structured approach in their establishment. If supported by competent teams, the availability of these tools can be an extremely effective factor.

There is a question that is often asked. It is related to the appropriate prioritization of the various projects of healthcare IT. In many industrialized countries, an error has often been made in the past. Information systems started with a focus on administrative aspects where the return on investment in terms of learning best practices, improving the quality of the care and controlling cost and resources were not the main priorities. The lesson learned for developing economies is to avoid repeating the same mistake.

## Conclusion

Our work is mostly focused on finding a model of HIS which is economically and locally acceptable to developing countries, especially in French speaking Africa, where our study took place.

Our study showed that developing countries constitute a brand new and wide open area of hospital information computerization. Multiple factors explain this situation. The exorbitant cost of proprietary commercial software is one component. A second reason is the information management culture of hospitals which has so far been concentrated on administrative and accounting tasks. This view is outdated. The quality care processes should be based on medical knowledge (quality improvement of individual players) and quality system processes (collective improvement of practice). In both cases, the computerized information system is the first cornerstone of the strategy. Although this condition is necessary, it is not sufficient. Another cornerstone is the local implementation strategy which requires the development of a strong level of expertise. Given the low cost of software labor, the available funds must be invested in developing this expertise. This strategy reduces the risk of failure and enables in all cases to capitalize the knowledge in the country and the hospital.

Open source is a disruptive paradigm that has the potential to improve the delivery of care and outcome in healthcare. Therefore, we encourage healthcare agencies in developing countries to engage and adopt quality Open Source software that uses international information technology standards.

The next step in our study will focus, on the complete integration of the billing module into the MediBoard application. After an appropriate period of use in Mali, we will assess the system's impact on quality of hospital care, as well as users' satisfaction.

## Competing interests

The authors declare that they have no competing interests.

## Authors' contributions

FM designed the research project. FM, OB, COB and JCD conducted the research. FM and OB developed the perspective of the concept. COB wrote the first draft of the manuscript. COB and SC brought the knowledge of local realities in the developing countries. JCD, COB, OB and FM conducted the literature review. FM wrote the final French version of the paper. OB and COB improved the English literature. All authors participated in the review of the manuscript, corrected it and approved it.

## Pre-publication history

The pre-publication history for this paper can be accessed here:

http://www.biomedcentral.com/1472-6947/10/22/prepub

## References

[B1] Amarasingham R Plantinga L Diener-West M Gaskin DJ Powe NR Clinical information technologies and inpatient outcomes. A multiple hospital study. Arch Int Med 2009 169 2 pp108 114 10.1001/archinternmed.2008.520 19171805

[B2] Heeks R 'Health Information Systems: Failure, Success And Improvisation' International Journal of Medical Informatics 2006 75 2 125 137 1386-5056 10.1016/j.ijmedinf.2005.07.024 16112893

[B3] Lamouche D L'open source: un levier stratégique de flexibilité et de souveraineté Editorial http://www.Bull.com/fr/libre consulté le 19/02/09

[B4] Tolentino H Marcelo A Marcelo P Maramba I Linking Primary Care Systems and Public Health Vertical Programmes in the Philippines: An Open-source Experience AMIA 2005 311 315 PMC1560490 16779052

[B5] Yu P De Courten M Pan E Galea G Pryor J The Developement and evaluation of PDA-based method for public health surveillance data collection in developing countries JM Inform 2009 78 8 532 42 Epub 2009 Apr15 10.1016/j.ijmedinf.2009.03.002 19369114

[B6] McDonald CJ Gunther S Barnes M Open Source sofware in medical informatics - why, how and what IJMI 2003 69 175 184 10.1016/s1386-5056(02)00104-1 12810121

[B7] Forrester consulting Open source software's expanding rôle in the enterprise Report 2007 Forrester Research Inc.,400 Technology Square, Cambridge MA 02139 USA

[B8] Categories of free and non-free software: Proprietary software http://www.gnu.org/philosophy/categories.html#ProprietarySoftware Last visit 10 January

[B9] 11 projets open source certifiés comme sûrs http://www.securityvibes.com/securite-open-source-audit-faille-coverity-acz-news-886.html last visit 29 January 2010

[B10] Yi Q Hoskins RE Hillringhouse E Integrating open-source technologies to build low-cost information systems for improved access to public health data. International Journal of Health Geographics 2008 7 29 10.1186/1476-072X-7-29 PMC2432052 18541035

[B11] Patel A O'Brien R Jones P Quintana Y Interorability of Open Source Medical Record Systems AMIA 2003 Symposium Proceedings 965 PMC1480230 14728469

[B12] Fraser H Biondich P Moodley D Choi S Mamlin B Szolovits P Implementing electronic medical record systems in developing countries Informatics in Primary Care 2005 13 83 95 15992493 10.14236/jhi.v13i2.585

[B13] Vanmeulebrouk B Rivett U Richetts A Loudon Open source GIS for HIV/AIDS management International Journal of Health Geographics 2008 7 58 10.1186/1476-072X-7-53 18945338 PMC2584066

[B14] Open Health Tools http://www.openhealthtools.org site visite le 9 mars 2009

[B15] Accueil http://www.mediboard.org/public/tiki-index.php visité le 9 Mars 2009

[B16] Hospital OS Software http://www.hospital-os.com/en/hospitalOS.php visité le 9 Mars 2009

[B17] Care2x http://www.care2x.org/visité le 9 Mars 2009

[B18] E-health Resolution World Health Assmbly 2005 http://www.who.int/healthacademy/news/eHealth_EB_Res-fr.pdf last visit December 2009

[B19] Hersh W Margolis A Quiros F Otero P Determining Health Informatics Workforce Needs in Developing Economies Making: the eHealth connection, Bellagio 2008

[B20] Thierney WM Beck EJ Gardner RM Musick B Shields M Shiyonga NM Spohr MH Viewpoint: A Pragmatic Approach to Contructing a Minimum data set for for care of patients with HIV/AIDS in developing countries J Am Med Inform Assoc 2006 13 3 253 60 10.1197/jamia.M2005 16501175 PMC1513663

[B21] Paré G Wybo MD Delannoy C Barriers to Open Source Software Adoption in Quebec's Health Care Organization J Med Syst 33 1 1 7 10.1007/s10916-008-9158-4 19238891

